# Time to discontinuation of atypical versus typical antipsychotics in the naturalistic treatment of schizophrenia

**DOI:** 10.1186/1471-244X-6-8

**Published:** 2006-02-21

**Authors:** Haya Ascher-Svanum, Baojin Zhu, Douglas Faries, Ron Landbloom, Marvin Swartz, Jeff Swanson

**Affiliations:** 1 Lilly Research Laboratories, Eli Lilly and Company, Indianapolis, Indiana, USA; 2 Department of Psychiatry and Behavioral Sciences, Duke University Medical Center, Durham, North Carolina, USA

## Abstract

**Background:**

There is an ongoing debate over whether atypical antipsychotics are more effective than typical antipsychotics in the treatment of schizophrenia. This naturalistic study compares atypical and typical antipsychotics on time to all-cause medication discontinuation, a recognized index of medication effectiveness in the treatment of schizophrenia.

**Methods:**

We used data from a large, 3-year, observational, non-randomized, multisite study of schizophrenia, conducted in the U.S. between 7/1997 and 9/2003. Patients who were initiated on oral atypical antipsychotics (clozapine, olanzapine, risperidone, quetiapine, or ziprasidone) or oral typical antipsychotics (low, medium, or high potency) were compared on time to all-cause medication discontinuation for 1 year following initiation. Treatment group comparisons were based on treatment episodes using 3 statistical approaches (Kaplan-Meier survival analysis, Cox Proportional Hazards regression model, and propensity score-adjusted bootstrap resampling methods). To further assess the robustness of the findings, sensitivity analyses were performed, including the use of (a) only 1 medication episode for each patient, the one with which the patient was treated first, and (b) all medication episodes, including those simultaneously initiated on more than 1 antipsychotic.

**Results:**

Mean time to all-cause medication discontinuation was longer on atypical (N = 1132, 256.3 days) compared to typical antipsychotics (N = 534, 197.2 days; p < .01), and longer on atypicals compared to typicals of high potency (N = 320, 187.5 days; p < .01), medium potency (N = 140, 213.5 days; p < .01), and low potency (N = 74, 208.7 days; p < .01). Among the atypicals, only clozapine, olanzapine, and risperidone had significantly longer time to all-cause medication discontinuation compared to typicals, regardless of potency level, and compared to haloperidol with prophylactic anticholinergic treatment. When compared to perphenazine, a medium-potency typical antipsychotic, only clozapine and olanzapine had a consistently and significantly longer time to all-cause medication discontinuation. Results were confirmed by sensitivity analyses.

**Conclusion:**

In the usual care of schizophrenia patients, time to medication discontinuation for any cause appears significantly longer for atypical than typical antipsychotics regardless of the typical antipsychotic potency level. Findings were primarily driven by clozapine and olanzapine, and to a lesser extent by risperidone. Furthermore, only clozapine and olanzapine therapy showed consistently and significantly longer treatment duration compared to perphenazine, a medium-potency typical antipsychotic.

## Background

Schizophrenia is often a severe and persistent mental illness, characterized by cognitive deficits, thought disorganization, mood abnormalities, and multiple functional deficits. Expert consensus guidelines for the treatment of schizophrenia [1] have identified psychosocial interventions and continuous antipsychotic medications as core treatment modalities. Interruptions in antipsychotic therapy have been shown to diminish treatment effectiveness [2-4] and increase the risk of hospitalization [4-6], even when the medication is interrupted for as little as 10 days [3]. Despite consistent treatment recommendations and recognition of the importance of continuous medication treatment, only about 50% of schizophrenia patients are adherent to antipsychotic medication regimens [7].

Consensus on the importance of continuous treatment with antipsychotics has been accompanied, however, by an ongoing debate over whether the newer "atypical" antipsychotics are superior to the older "typical" antipsychotics in the treatment of schizophrenia [8-13]. Some have reported comparable efficacy [12] and questioned the claims of superior efficacy of atypicals relative to typicals [8,12,13] because clinical trials have often established efficacy of the atypicals in comparison to haloperidol, a high-potency typical antipsychotic known to have pronounced treatment-emergent extrapyramidal symptoms (EPS) that may contribute to poorer treatment outcomes. Some researchers have claimed [8] that atypicals are of comparable efficacy to typical antipsychotics of medium or low potency, because lower-potency antipsychotics have a more favorable EPS profile, and thus provide greater tolerability and better outcomes. Others also reported [12] that olanzapine is not more efficacious than haloperidol when haloperidol is provided with prophylactic anticholinergic treatment to help ameliorate EPS.

Time to all-cause medication discontinuation has been recognized as an important global index of antipsychotic effectiveness, because it reflects the judgment of both patients and clinicians on the medication's effectiveness, safety, and tolerability [14]. This global proxy measure of medication effectiveness is the primary outcome measure in the National Institute of Mental Health Clinical Antipsychotic Trials of Intervention Effectiveness (CATIE) project [14], an important 18-month, randomized, double-blind study comparing 1 typical antipsychotic (perphenazine) and 4 atypical antipsychotics (olanzapine, risperidone, quetiapine, and ziprasidone) in the treatment of schizophrenia in the United States. The CATIE trial concluded that olanzapine was the most effective in terms of discontinuation rates and time to discontinuation [14]. The median time to discontinuation of treatment for any cause was significantly longer in the olanzapine group (9.2 months) than in the quetiapine (4.6 months, p < .001) or risperidone group (4.8 months, p = .002). The median time to all-cause treatment discontinuation was also longer for olanzapine compared to perphenazine (5.6 months, p = .021) and ziprasidone (3.5 months, p = .028), but these differences lost their statistical significance following adjustments for multiple comparisons. It is important to note that for various reasons [14], the specific comparisons involving perphenazine and ziprasidone had a lower statistical power to identify group differences in treatment discontinuation (statistical power of 76% and 58% for perphenazine and ziprasidone, respectively, rather than 85%). It is unclear whether findings from CATIE, a randomized, double blind study, may generalize to open-label, naturalistic treatment in usual care settings. The objective of this study is to compare atypical and typical antipsychotics on time to all-cause medication discontinuation in the usual care of patients with schizophrenia spectrum disorders. We used data from a large, prospective, non-randomized, non-interventional, multisite, naturalistic study of schizophrenia patients in the United States. Using time to all-cause medication discontinuation for 1 year following medication initiation, we first compared 5 atypical antipsychotics (singly and combined) with typical antipsychotics of high-, medium-, or low-potency level. Next, we compared the atypicals (singly and combined) with 2 specific typical antipsychotics: (a) perphenazine, a medium-potency typical antipsychotic, and (b) haloperidol with prophylactic anticholinergic agents. Consistent with meta-analytical findings from randomized clinical trials (RCTs) [9-11], we hypothesized that during treatment of schizophrenia patients in "real-world settings," time to medication discontinuation for any cause is longer for patients treated with atypical than typical antipsychotics, regardless of potency level, and that time to all-cause medication discontinuation will also differ among atypical antipsychotics compared to typical agents.

## Methods

### Subjects and study design

We used data from the U.S. Schizophrenia Care and Assessment Program (US-SCAP), a large (N = 2327), non-randomized, multicenter, 3-year, prospective, naturalistic study of patients treated for schizophrenia spectrum disorders. The study was conducted between July 1997 and September 2003, and its goal was to understand the treatment of schizophrenia patients in usual care settings in the United States [15-19]. Patients were recruited from diverse geographical areas, including the Northeast, Southwest, Mid-Atlantic, and West. The 6 participating regional sites represented large systems of care, including community mental health centers, university health care systems, community and state hospitals, and the Department of Veterans Affairs Health Services. Each participating site had to provide open and unrestricted access to all atypical antipsychotics available in the U.S. during the study period (clozapine, risperidone, olanzapine, quetiapine, and ziprasidone). Participants were adults -18 years and older with no upper age limit – treated for schizophrenia, schizoaffective, or schizophreniform disorders, based on DSM-IV criteria, were geographically and ethnically diverse, and represented treatment in large systems of care. Patients were excluded if they were unable to provide informed consent or had participated in a clinical drug trial within 30 days prior to enrollment. Institutional Review Board (IRB) approval was received at each regional site prior to initiation of the study, and informed consent was received from all participants.

The study enrollment process used 2 recruitment strategies simultaneously: random selection of outpatients from site medical information rosters of active clients, and sequential screening of inpatient admissions. Outpatients were selected using de-identified lists of adults currently receiving services at the site with diagnosis of schizophrenia spectrum disorders. Patient medication information was excluded. Using a random number generator, outpatients were selected at random. The de-identified lists were refreshed every 6 months to replace the patients that had been recruited, until each site reached the enrollment target (400 patients). Of 3332 patients who met the minimal inclusion criteria noted above, 2327 (69.8%) enrolled, 765 (23.0%) refused, and 240 (7.2%) were not enrolled due to other reasons. This screening information pertains to inpatient and outpatients, as it was not collected separately by inpatient/outpatient status. Almost all study participants were outpatients at enrollment (2175/2327 or 93.5%). Of 2327 participants, most completed 1 year of follow-up (78.1%), with fewer completing 2 years (69.6%), and 3 years (65.2%). Enrollment was not contingent upon being treated with a specific antipsychotic or with any medication. Patients could continue with medications they received prior to enrollment for as long as necessary, and decisions about medication changes, if any, reflected those made by physicians and their patients, as they naturally occur in usual practice.

### Analytical sample

**Figure 1:**
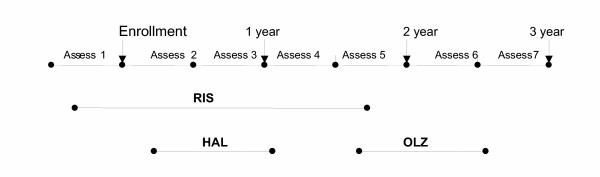
**Treatment-episodes approach**. Abbreviations: RIS, risperidone; OLZ, olanzapine; HAL, haloperidol. Using the treatment episode approach, a patient may have initiated several treatment episodes over the course of this 3-year study. This figure shows an example of the treatment pattern of a patient with 3 treatment episodes. Patients were administered study measures at the end of each 6-month assessment period.

The analytical sample for the present study included treatment episodes of patients who were initiated at any time during the 3-year study or the 4 months prior to enrollment on any oral antipsychotic and had at least 1 year of follow-up in the study following initiation on the medication. Initiation was defined as the start of an antipsychotic that was not prescribed during the previous 60 days or more. Using an episode-of-care approach provides comprehensive information about the multiple courses of treatment per participant in the longitudinal treatment of the illness [20]. Figure 1 illustrates the treatment-episodes approach, and the potential for overlap between treatment episodes for any given patient during the 3-year study. A total of 1666 treatment episodes of 1028 patients met these criteria. The 1666 treatment episodes included 1132 on atypical antipsychotics and 534 on typical antipsychotics. The atypical treatment group was comprised of clozapine (N = 114), olanzapine (N = 465), risperidone (N = 350), quetiapine (N = 178), and ziprasidone (N = 25). The typical antipsychotic treatment group included typicals of high potency (N = 320, haloperidol and fluphenazine); typicals of medium potency (N = 140, loxapine, molindone, perphenazine, thiothixene, and trifluoperazine); and typicals of low potency (N = 74, chlorpromazine, mesoridazine, promethazine, and thioridazine). The medium-potency typical antipsychotic group included perphenazine (N = 41), and the high-potency typical antipsychotic group included haloperidol with prophylactic anticholinergics (N = 114). Prophylactic use was defined as a prescription for an anticholinergic medication written on the same date as, or 1 day later than, the initiation date of haloperidol.

### Measures

The primary outcome measure was time to all-cause medication discontinuation. This measure assessed the number of consecutive days from initiation of the index antipsychotic to the start of the first medication gap larger than 30 consecutive days during the first year following initiation. Previous research has established the absence of target medication for more than 14 days as a valid indicator of drug discontinuation [4,21]. We opted to be more conservative and used a larger gap to denote medication discontinuation [22]. A medication gap could have resulted from any number of events, including switching to another antipsychotic or discontinuation of the antipsychotic drug. The manner in which we determined medication gap size was based on periodic (every 6 months) and systematic reviews of medical records using an abstraction form developed for this study. Trained and annually certified examiners used this form to collect medication prescription information, which was used to identify an absence of a prescription for each index antipsychotic for more than 30 consecutive days.

At enrollment and at 12-month intervals thereafter, participants were assessed with various standard psychiatric measures, but these planned study assessments were not set to coincide with time of initiation or discontinuation of any medication.

### Statistical analysis

Treatment group comparisons on time to all-cause medication discontinuation were first made between the atypicals as a group versus typicals as a group, then between the atypicals (singly and combined) and typicals of high potency, medium potency, and low potency levels. In addition, the atypicals were compared (singly and combined) with perphenazine, and then with haloperidol with prophylactic anticholinergics.

Data analyses posed a number of analytical challenges that were identified and addressed using the following approaches:

#### a) Potential selection bias due to non-randomization

Treatment assignments in usual practice tend to reflect patient's illness profile, prior medication use pattern, and physician's medication preferences. To help address potential selection bias of the treatment groups, we identified, *a priori*, a set of available covariates that were used in the Cox Proportional Hazards regression model (covariates are listed later in this section). In addition, we used propensity scores adjustment to balance the characteristics of the treatment group comparisons [23].

#### b) Potential "period bias" due to differential time of medication introduction in the U.S

This issue was especially pertinent for antipsychotics that were launched later (quetiapine and ziprasidone). To help address potential "period bias," we included time to initiation on the index antipsychotic from the starting date of the US-SCAP study as a covariate in the Cox Proportional Hazards regression model, and in the propensity score method.

#### c) Skewed data distribution

The distribution of data on time to all-cause medication discontinuation was found to be skewed. To address this issue, bootstrapping method after propensity score adjustment was used to compare the mean difference between treatment groups on time to all-cause discontinuation [24]. Other statistical methods (Kaplan-Meier survival analysis and Cox Proportional Hazards regression models) that do not impose symmetry were used to confirm the findings.

#### d) Potential bias due to episode approach

Use of treatment episode as the unit of analysis may create bias, because 1 patient may have been initiated on several medications over time, thus contributing multiple episodes. To address this issue, we performed a sensitivity analysis, using each patient's first episode only.

#### e) Potential "sponsor-related physician bias"

Favorable perceptions of the pharmaceutical company sponsoring the study may create preference of the sponsor's medication (olanzapine in this case) by participating physicians over other antipsychotics. Although this potential bias cannot be ruled out by statistical methods, we attempted to address physicians' potential preference to switch patients to olanzapine soon after patients' enrollment in the study. To that end, we performed an additional sensitivity analysis in which the time from patient's enrollment in US-SCAP to the time of patient's initiation on the index medication was used as a covariate in the Cox Proportional Hazards regression model. We hypothesized that if "sponsor-related physician bias" were present, physicians would have quickly switched patients from their current antipsychotic to olanzapine rather than to risperidone (olanzapine, risperidone, and clozapine were available for all patients at the time of enrollment, unlike quetiapine and ziprasidone that were not yet available to early study enrollees).

#### f) Lower statistical power due to smaller sample size of the ziprasidone treatment group

The smaller sample is likely due to various factors, including later time to launch of ziprasidone in the U.S., and the drugs' share of the total antipsychotic prescription market. We were unable to statistically address the small sample size problem and suggest, therefore, that the ziprasidone-related findings be interpreted with caution.

To address these multiple analytical challenges, we used 3 separate statistical approaches for the main analysis and performed 5 additional sensitivity analyses. For the main analysis, all treatment group comparisons were made using (a) Kaplan-Meier survival analysis (log-rank test), (b) Cox Proportional Hazards regression model, and (c) propensity score-adjusted bootstrap resampling method with 1000 iterations. To address possible selection bias in patients' assignment to medication groups in this non-randomized study, we selected, *a priori*, a set of available variables that were used to statistically adjust for possible group differences at the time of initiation on the index medication (in the Cox Proportional Hazards regression model, and the propensity score method). Most of these socio-demographic and treatment variables have been previously reported to be related to nonadherence with antipsychotic medication in the treatment of schizophrenia [25]. The covariates included age, gender, ethnicity, comorbid diagnosis of substance use disorder, psychiatric hospitalization in the 60 days prior to medication initiation, time elapsed between medication initiation and beginning of the US-SCAP study (01/07/1997), enrollment site (1–6), schizoaffective disorder diagnosis (yes/no), and duration on any antipsychotic in the 60 days prior to initiation on the index antipsychotic.

The third main statistical approach used a propensity score-adjusted bootstrap resampling method. To perform this combined approach, we first calculated propensity scores for each patient in each pairwise comparison using a binary logistic model based on the covariates just presented. Next, the propensity scores were divided into bins based on quintiles of the propensity score distribution. We then used bootstrap resampling evenly across bins to produce a sample from which we could compute a mean treatment difference in days to all-cause medication discontinuation that was adjusted for the listed covariates. This process was repeated 1000 times to create a distribution of days to all-cause discontinuation from which non-parametric p-values for the mean treatment differences were obtained.

In addition, 5 sensitivity analyses were performed (using the Cox Proportional Hazards regression model with the identified covariates) to address other potential sample selection biases. In the first sensitivity analysis, only the first episode for each patient was used (N = 1028). In the second sensitivity analysis, all treatment episodes were included (N = 1703, 1166 on atypicals, 537 on typicals), thus adding treatment episodes on which patients were simultaneously initiated on more than one antipsychotic (adding 37 to the 1666 treatment episodes used in the main analysis). The third sensitivity analysis assessed "sponsor-related physician bias" by including the new covariate just described: the time elapsed between medication initiation and the patient's enrollment date in the study. This covariate replaced another covariate used in the main analyses (time elapsed between medication initiation and beginning of US-SCAP). The fourth sensitivity analysis assessed the robustness of the medication gap used to signal medication discontinuation (>30 days) by repeating the analyses with the more customary "longer-than-14-day" medication gap. The fifth and last sensitivity analysis was an intent-to-treat (ITT) analysis, which unlike previous analyses also included initiators on the index drug who did not have data for at least 1 year following initiation. The ITT approach was added to help address the possibility that the study findings are limited to "1-year study completers," and included 2488 treatment episodes (1722 on atypicals, 766 on typicals).

Although statistical tests were 2-tailed and significance was set at .05 alpha level, we also pursued corrections for multiple comparisons. This was done because we conducted a large number of statistical comparisons, and a few of them could have provided significant findings by chance. Consistent with the CATIE trial [14], we used a stepwise (gatekeeper) approach by means of a Hochberg adjustment for multiple comparisons [26]. First, we tested the differences between atypicals and typicals (Step 1). When significance (p < .05) was found, we then tested each atypical versus typicals as a class (5 comparisons, p = .01 set as the threshold) (Step 2). Next, when significance was found in Step 2 for a specific atypical, that atypical was tested versus typicals of high, medium, and low potency (3 comparisons, p = .017 set as the threshold) (Step 3). Lastly (Step 4), once significant findings were found in Step 3 for atypicals versus high-potency typicals, we compared each atypical to haloperidol with prophylactic anticholinergics; and once significant findings were found in Step 3 for the atypicals versus medium-potency typicals, we compared each atypical to perphenazine (5 comparisons, p = .017 set as the threshold).

## Results

### Patient characteristics

This study included 1666 treatment episodes of 1028 patients who were initiated on an oral antipsychotic and had at least 1 year of follow-up in the study following medication initiation. Of 1028 patients, 60% had 1 treatment episode, 26% had 2 treatment episodes, 9% had 3 treatment episodes, and 5% had 4 or more treatment episodes.

At enrollment (Table 1), the 1028 patients were 40.8 years old on average (age range 18–78) and were likely to be male (59.2%) and White (51.9%). Most patients (81.2%) had health care coverage by Medicaid or Medicare, or both, and 7.5% were uninsured. Medicaid and Medicare are public agencies that provide U.S. government and state-funded health insurance coverage. Medicaid is a program sponsored by the U.S. federal government and administered by states to provide health-related services to low-income individuals. Medicare is a U.S. federal health insurance program for people age 65 and older and for individuals with disabilities. On average, patients were moderately and chronically ill with mild depressive symptoms, poor quality of life, and 20.7 years of illness duration. About a third were diagnosed with schizoaffective disorder and 28.8% with comorbid substance use disorder. About one-fourth (25.6%) had a psychiatric hospitalization at enrollment or during the 6 months prior to enrollment.

**Table 1:** Patient characteristics at enrollment (n = 1028)

Characteristic	Value
Age, years, mean (SD)	40.8 (11.0)
Male gender, % (n)	59.2 (609)
Ethnicity, % (n)	
White	51.9 (553)
Black	39.2 (403)
Other	8.9 (92)
Schizoaffective disorder, % (n)	34.3 (353)
Illness duration, mean (SD)	20.7 (11.6)
Coverage by Medicaid and/or Medicare, % (n)	81.2 (835)
No health insurance % (n)	7.5 (77)
Substance use disorder, % (n)	28.8 (296)
Psychiatric hospitalization at enrollment or in prior 6 months, % (n)	25.6 (263)
Quality of Life Scale, total score, mean (SD)	73.9 (19.4)
MADRS, mean (SD)	15.7 (10.7)
PANSS, total score, mean (SD)	73.2 (18.6)

Abbreviations: SD, standard deviation; MADRS, Montgomery-Asberg Depression Rating Scale; PANSS, Positive and Negative Syndrome Scale

Available patient characteristics at baseline – the time of initiation on the index drug – were fewer than characteristics assessed at the time of patients' enrollment in the study, because the assessments were not set to coincide with time of initiation or discontinuation of any medication. Baseline characteristics for the treatment groups prior to initiation are presented in Table 2. The treatment groups differed in age, gender, ethnicity, comorbid diagnosis of substance use disorder, psychiatric hospitalization in the 60 days prior to medication initiation, time elapsed between medication initiation and beginning of the US-SCAP study, schizoaffective disorder diagnosis, and duration on any antipsychotic in the 60 days prior to initiation on the index antipsychotic. As previously noted, these variables were used as covariates in the analyses.

The mean (median) daily dose of each antipsychotic medication group during the 1 year following its initiations, in mg/day, was: 364.4 (356.6) for clozapine; 14.0 (12.3) for olanzapine; 4.3 (4.0) for risperidone; 340.2 (297.3) for quetiapine; and 107.6 (113.0) for ziprasidone. In addition, the average daily dose was 14.8 (8.0) for perphenazine, and 10.4 (9.3) for haloperidol with prophylactic anticholinergic agents. The average daily doses were within standard treatment range as noted in the drugs' package inserts.

**Table 2:** Patient characteristics at the time of medication initiation

Variable	CLZ N = 114	OLZ N = 465	RIS N = 350	QUE N = 178	ZIP N = 25	PER N = 41	High N = 320	Med N = 140	Low N = 74	HAL+AC N = 114
Mean age, years	37.3	41.6	40.3	39.6	37.6	40.1	39.0	40.2	37.9	38.2
Male, %	57.0	60.2	54.6	47.2	24.0	56.1	58.8	60.0	64.9	57.0
African American, %	24.6	40.4	38.3	34.8	12.0	39.0	46.3	35.0	33.8	41.2
Substance use disorder, %	21.9	31.2	30.3	21.4	36.0	31.7	37.5	25.7	39.2	36.8
Psychiatric hospitalization in prior 2 months, %	43.0	23.4	31.2	20.2	20.0	19.5	42.5	32.1	31.1	45.6
Mean time from study start to initiation, days	7000.3	656.0	689.7	828.4	1403.6	635.8	604.6	646.8	622.1	612.7
Schizoaffective disorder, %	34.2	32.0	34.3	39.9	60.0	29.3	32.2	37.1	33.8	32.5
Mean time on any antipsychotic in prior 2 months, days	51.6	49.1	46.4	55.7	57.8	38.3	44.8	42.7	49.2	46.8

Abbreviations: CLZ, clozapine; OLZ, olanzapine; RIS, risperidone; QUE, quetiapine; ZIP, ziprasidone; PER, perphenazine; High, high-potency typical antipsychotics; Med, medium-potency typical antipsychotics; Low, low-potency typical antipsychotics; HAL+AC, haloperidol with prophylactic anticholinergic agents.

### Atypicals versus typicals

**Figure 2:**
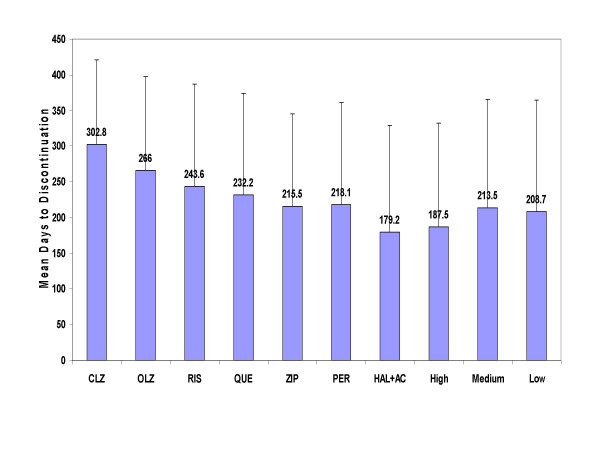
**Mean time to all-cause medication discontinuation for atypical and typical antipsychotics**. Abbreviations: CLZ, clozapine; OLZ, olanzapine; RIS, risperidone; QUE, quetiapine; ZIP, ziprasidone; PER, perphenazine; HAL+AC, haloperidol with prophylactic anticholinergic agents; High, high-potency typical antipsychotics; Med, medium-potency typical antipsychotics; Low, low-potency typical antipsychotics. Error bars represent standard deviations. Significant differences (p < .05) between high-potency typicals and CLZ, OLZ, RIS, QUE; between medium-potency typicals and CLZ, OLZ, RIS; between low-potency typicals and CLZ, OLZ, RIS; between perphenazine and CLZ, OLZ, RIS; and between haloperidol with prophylactic anticholinergics and CLZ, OLZ, and RIS. Comparisons made with Cox Proportional Hazards regression model adjusted for patients and prior treatment characteristics.

**Figure 3:**
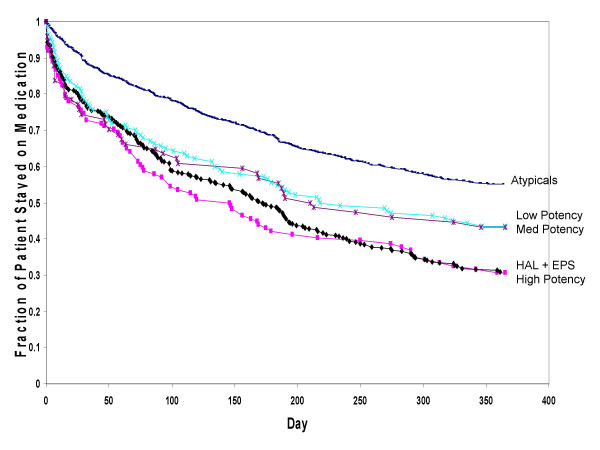
**Survival rates: time to all-cause medication discontinuation for atypicals versus typicals of different potency levels**. Abbreviation: HAL+AC, haloperidol with prophylactic anticholinergics. Significant differences (p < .05) between the combined atypicals versus typicals of high, medium, and low potency, and versus haloperidol with prophylactic anticholinergics, using Cox Proportional Hazards regression model adjusted for patients and prior treatment characteristics.

During the 1 year following medication initiation, the combined atypical antipsychotic group had significantly longer treatment duration (256.3 days, SD = 137.2) than the combined typical antipsychotic treatment group (197.2 days, SD = 148.6; p < .01; Table 3, Figure 2). Similarly, the combined atypical antipsychotic treatment group had significantly (p < .01) longer treatment duration compared to typical antipsychotics of high, medium, and low potency (Table 3). Survival curves are presented in Figure 3 for atypicals as a group versus typicals of high-, medium-, and low-potency levels.

**Figure 4:**
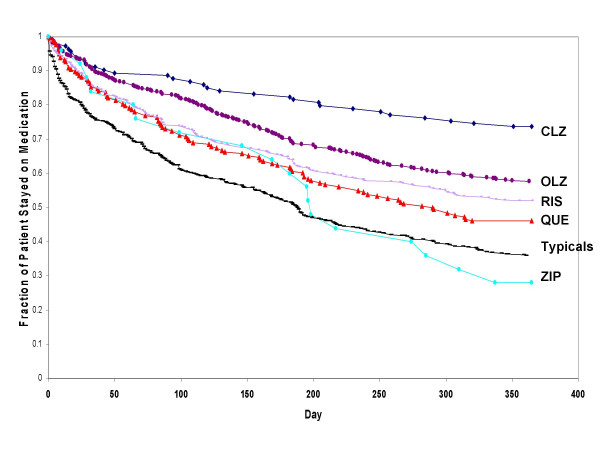
**Survival rates: time to all-cause medication discontinuation for clozapine, olanzapine, risperidone, quetiapine, ziprasidone versus typicals**. Abbreviations: CLZ, clozapine; OLZ, olanzapine; RIS, risperidone; QUE, quetiapine; ZIP, ziprasidone. Significant differences between CLZ, OLZ, and RIS versus the combined typicals at p < .05 using the Cox Proportional Hazards regression model adjusted for patients and prior treatment characteristics.

When comparing each of the atypicals to the typicals (combined), and to typicals of high-, medium-, or low-potency level (separately), only clozapine, olanzapine, and to a lesser extent risperidone were consistently associated with significantly longer time to medication discontinuation (p < .05; Table 3). Treatment with clozapine and olanzapine was associated with a significantly longer time to discontinuation compared to treatment with high-, medium-, or low-potency typical antipsychotics. Treatment with risperidone was significantly longer than typicals of high and medium potency but did not consistently differ from typicals of low-potency level. Treatment with quetiapine was significantly longer than with typicals of high potency, did not consistently differ from typicals (combined), and did not differ from typicals of medium- or low-potency levels. Treatment with ziprasidone did not significantly differ from typicals, regardless of potency level. Findings maintained their significance following correction for multiple comparisons (Steps 2 and 3). Figure 4 demonstrates that the likelihood of staying on an atypical antipsychotic compared to typical antipsychotics (combined) differed among the atypicals, such that only clozapine, olanzapine, and risperidone had consistently longer time to medication discontinuation compared to treatment with typical agents. Table 5 presents the hazard ratios (HR) and 95% confidence intervals of time to all-cause medication discontinuation of each atypical agent versus all the typicals using the Cox Proportional Hazards regression model. The HR varied greatly, ranging from highest (for clozapine, HR = 3.51) to lowest (for ziprasidone, HR = 0.84).

**Table 3:** Differences between atypical and typical antipsychotics of high, medium, and low potency on time to all-cause medication discontinuation

	**CLZ, OLZ, RIS, QUE, ZIP (combined)**	**CLZ**	**OLZ**	**RIS**	**QUE**	**ZIP**
**High**-potency typicals						
Log-rank test	**	**	**	**	**	NS
Cox PH model	**	**	**	**	*	NS
Propensity score bootstrap	**	**	**	**	*	NS
**Medium**-potency typicals						
Log-rank test	**	**	**	*	NS	NS
Cox PH model	**	**	**	*	NS	NS
Propensity score bootstrap	**	**	**	*	NS	NS
**Low**-potency typicals						
Log-rank test	**	**	**	NS	NS	NS
Cox PH model	*	**	*	*	NS	NS
Propensity score bootstrap	**	**	**	*	NS	NS
**All **typicals						
Log-rank test	**	**	**	**	**	NS
Cox PH model	**	**	**	**	NS	NS
Propensity score bootstrap	**	**	**	**	NS	NS

Abbreviations: CLZ, clozapine; OLZ, olanzapine; RIS, risperidone; QUE, quetiapine; ZIP, ziprasidone; NS, not significant *Significant at p < 0.05 level **Significant at p ≤ 0.01 level

**Table 4:** Differences between atypical antipsychotics and perphenazine on time to all-cause medication discontinuation

**Antipsychotic**	Statistical approach		
	
	Survival analysis, log-rank test	Cox PH model	Propensity score bootstrap
CLZ, OLZ, RIS, QUE, ZIP (combined) vs. PER	*	*	**
Clozapine vs. PER	**	**	**
Olanzapine vs. PER	**	**	**
Risperidone vs. PER	NS	*	NS
Quetiapine vs. PER	NS	NS	NS
Ziprasidone vs. PER	NS	NS	NS

Abbreviations: CLZ, clozapine; OLZ, olanzapine; RIS, risperidone; QUE, quetiapine; ZIP, ziprasidone; NS, not significant; PER, perphenazine.

*Significant at p < 0.05 level

**Significant at p ≤ 0.01 level

**Table 5:** Hazard ratios of time to medication discontinuation for atypical antipsychotics versus all typicals, versus perphenazine and versus haloperidol with prophylactic anticholinergics

**Treatment groups**	**Hazard ratios (95% confidence interval)**
Clozapine vs. All Typicals	3.51** (2.39, 5.18)
Olanzapine vs. All Typicals	1.74** (1.45, 2.08)
Risperidone vs. All Typicals	1.56** (1.29, 1.88)
Quetiapine vs. All Typicals	1.24** (0.97, 1.58)
Ziprasidone vs. All Typicals	0.84 (0.49, 1.44)
	
Clozapine vs. Perphenazine	3.25** (1.70, 6.21)
Olanzapine vs. Perphenazine	1.76** (1.15, 2.70)
Risperidone vs. Perphenazine	1.60* (1.04, 2.46)
Quetiapine vs. Perphenazine	1.06 (0.64, 1.77)
Ziprasidone vs. Perphenazine	0.76 (0.25, 2.33)
	
Clozapine vs. Haloperidol +	3.61** (2.26, 5.78)
Olanzapine vs. Haloperidol +	1.89** (1.43, 2.51)
Risperidone vs. Haloperidol +	1.71** (1.29, 2.25)
Quetiapine vs. Haloperidol +	1.33 (0.94, 1.88)
Ziprasidone vs. Haloperidol +	0.71 (0.32, 1.58)

Abbreviations: Haloperidol + denotes the use of haloperidol with prophylactic anticholinergic agent.

Prophylactic use of anticholinergics is defined as a prescription for haloperidol and an anticholinergic on the date haloperidol was initiated, or 1 day later.

*Significant at p < 0.05 level

**Significant at p ≤ 0.01 level

### Atypicals versus perphenazine

**Figure 5:**
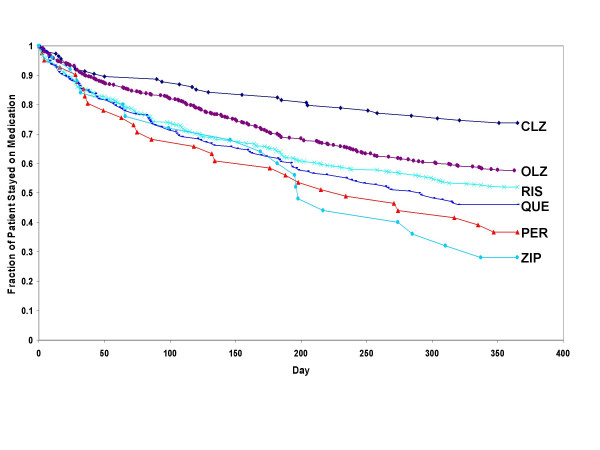
**Survival rates: time to all-cause medication discontinuation for clozapine, olanzapine, risperidone, quetiapine, ziprasidone versus perphenazine**. Abbreviations: CLZ, clozapine; OLZ, olanzapine; RIS, risperidone; QUE, quetiapine; ZIP, ziprasidone; PER, perphenazine. Significant treatment group differences between CLZ, OLZ, and RIS versus perphenazine at p < .05, using Cox Proportional Hazards regression model adjusted for patients and prior treatment characteristics.

The combined atypical antipsychotic treatment group had a significantly longer time to medication discontinuation compared to treatment with perphenazine, using the 3 statistical methods (Table 4, Figure 2). These findings were driven primarily by clozapine and olanzapine, as treatment with risperidone did not differ from perphenazine on 2 of the 3 statistical methods, whereas treatment with quetiapine or ziprasidone did not significantly differ from perphenazine on any of the 3 statistical methods. Figure 5 presents the probability of discontinuing the medication during the 1 year following initiation for each atypical antipsychotic compared to perphenazine, and Table 5 presents the hazard ratios (HR) and 95% confidence intervals of time to all-cause medication discontinuation of each atypical agent versus perphenazine using the Cox Proportional Hazards regression model. The HR varied greatly, ranging from highest (for clozapine, HR = 3.25) to lowest (for ziprasidone, HR = 0.76). Following correction for multiple comparisons, clozapine and olanzapine maintained their initial significant differences. The comparison between perphenazine and risperidone lost its significance (p = 0.03, required p value = 0.017), while comparisons with quetiapine and ziprasidone were unchanged (non-significant).

### Atypicals versus haloperidol with prophylactic anticholinergics

As presented in Table 6, the combined atypical antipsychotic treatment group had a significantly longer time to medication discontinuation compared to treatment with haloperidol with prophylactic anticholinergic agents, using the 3 statistical approaches.

**Table 6:** Differences between atypicals and haloperidol with prophylactic anticholinergic agents on time to all-cause medication discontinuation

**Antipsychotic**	Statistical approach		
	
	Survival analysis, log-rank test	Cox PH model	Propensity score bootstrap
CLZ, OLZ, RIS, QUE, ZIP (combined) vs. HAL+	**	**	**
Clozapine vs. HAL+	**	**	**
Olanzapine vs. HAL+	**	**	**
Risperidone vs. HAL+	**	**	**
Quetiapine vs. HAL+	**	NS	NS
Ziprasidone vs. HAL+	NS	NS	NS

Abbreviations: CLZ, clozapine; OLZ, olanzapine; RIS, risperidone; QUE, quetiapine; ZIP, ziprasidone; NS, not significant; HAL+ denotes the use of haloperidol with prophylactic anticholinergic agent.

*Significant at p < 0.05 level

**Significant at p ≤ 0.01 level

Prophylactic use of anticholinergics is defined as a prescription for haloperidol and an anticholinergic on the date haloperidol was initiated, or 1 day later.

These findings were driven primarily by clozapine, olanzapine, and risperidone, as quetiapine did not consistently differ from haloperidol with prophylactic anticholinergics, and treatment with ziprasidone did not significantly differ from haloperidol with prophylactic anticholinergics on any of the 3 statistical approaches. Table 5 presents the hazard ratios and 95% confidence intervals for time to medication discontinuation of each atypical agent versus haloperidol with prophylactic anticholinergic agents, using the Cox Proportional Hazards regression model. The HR ranged from a highest for clozapine (HR = 3.61) to the lowest for ziprasidone (HR = 0.71). Findings were unchanged following correction for multiple comparisons (Step 4).

The average time to all-cause medication discontinuation for each atypical antipsychotic, for perphenazine, and for haloperidol with prophylactic anticholinergics are presented with standard deviation error bars in Figure 2. Findings illustrate the ranking of the antipsychotics in the following descending order: clozapine, olanzapine, risperidone, quetiapine, perphenazine, ziprasidone, and haloperidol with prophylactic anticholinergics.

The proportion of treatment episodes that were at least 1-year long (conceptually similar to 1-year completer rates in RCTs) was 73.7% for clozapine, 57.6% for olanzapine, 52.0% for risperidone, 46.1% for quetiapine, 28.0% for ziprasidone, 30.9% for typicals of high-potency level, 43.6% for typicals of medium-potency level, 43.2% for typicals of low-potency level, 36.6% for perphenazine, and 30.7% for haloperidol with prophylactic anticholinergics.

### Sensitivity analyses

Results were essentially unchanged when the Cox Proportional Hazards regression model was repeated (a) using 1 medication episode per patient (n = 1028), the one with which the patient was treated first, (b) using 1703 treatment episodes, including episodes where patients simultaneously initiated 2 or more treatment episodes, (c) using the time elapsed between medication initiation and patients' enrollment in the study, as a proxy for potential "sponsor-related physician bias," (d) using a medication gap >14 days to assess robustness of the >30-day medication gap used to signal medication discontinuation, and (e) using ITT analysis, with 2488 treatment episodes, thus not requiring initiators to have continued in the study for at least 1 year following medication initiation.

Only 1 of the 5 sensitivity analyses, the one using medication gap >14 days, provided different results on 2 specific analyses. These analyses indicated that clozapine, olanzapine, risperidone, and quetiapine significantly (p < .05) differ from the combined typical antipsychotic group, and from haloperidol with prophylactic anticholinergics (quetiapine did not significantly differ from these 2 treatment groups in the main analyses).

## Discussion

Consistent with findings from meta-analyses of randomized, double-blind clinical trials [9-11], this large naturalistic observational study found significant differences between atypical and typical antipsychotics in the treatment of schizophrenia. The present findings may help extend and complement previous observations on treatment duration in RCTs to treatment in usual clinical practice, using a clinically meaningful proxy measure of medication acceptability that appears to capture the patient and clinician judgments of the medication, including symptomatic response, safety, and tolerability [14]. Longer treatment duration was previously shown to be associated with a lower risk of psychotic relapse and psychiatric hospitalization [3-5,14,27], thus likely to decrease patients' personal burden and lower the economic costs for acute care in the mental health system [6]. Longer treatment duration was also found to be associated with greater symptom improvement [28] and better functional outcomes in the treatment of patients with schizophrenia [29].

More notably, this naturalistic study presents novel and clinically important information about differences in treatment duration between specific atypicals and specific typicals (of high-, medium-, and low-potency levels; perphenazine, and haloperidol with prophylactic anticholinergic agents). Findings demonstrate that compared to typicals, the atypicals are not a homogeneous group in usual clinical practice. Remarkably, not all the 5 studied atypicals differentiated significantly and consistently from typicals of various potency levels, as findings were primarily driven by clozapine and olanzapine, and to a lesser extent by risperidone. Furthermore, only clozapine and olanzapine therapy showed consistently and significantly longer treatment duration compared to perphenazine, a medium-potency typical antipsychotic medication.

This study has also shown that some but not all atypical antipsychotics are associated with significantly longer treatment duration compared to haloperidol, when haloperidol is provided with prophylactic anticholinergics. Specifically, only clozapine, olanzapine, and risperidone were found to be associated with significantly longer treatment duration compared to haloperidol with prophylactic anticholinergic agents.

Current findings are consistent with several core findings from the CATIE trial [14]. Despite substantial differences in design and methodology between CATIE and US-SCAP (e.g., CATIE is a randomized, double blind trial and US-SCAP is non-randomized and non-interventional), both studies found atypical antipsychotics to be a heterogeneous group on time to all-cause medication discontinuation. When excluding clozapine, which was not included in Phase 1 of CATIE, both studies found olanzapine-treated patients to have the longest time to medication discontinuation, followed in descending order by risperidone, quetiapine, and ziprasidone. In both studies, treatment with olanzapine, but not with risperidone, quetiapine, or ziprasidone, was associated with significantly longer time to all-cause medication discontinuation when compared with perphenazine. The statistically significant difference versus perphenazine found in CATIE (p = .021) lost, however, its significance following corrections for multiple comparisons (required p value ≤ .017). However, when similar stepwise corrections for multiple comparisons were performed in the US-SCAP analyses, the findings were essentially unchanged, still favoring olanzapine over perphenazine. Current findings suggest that if time to all-cause medication discontinuation in usual care were to reflect medication effectiveness as it does in CATIE [14], than the effectiveness of clozapine and olanzapine relative to perphenazine, and the effectiveness of clozapine, olanzapine, and risperidone relative to haloperidol, may not be overestimated in usual care settings.

Our findings of differential treatment duration in usual care between typicals and atypicals are also consistent with previous reports of a lower rate of treatment failure and longer time to drug discontinuation [11,30-33] among schizophrenia patients treated with atypicals compared with typicals. Generally, there appears to be a 2-fold increased risk of drug discontinuation with typicals compared with atypicals [34]. Current findings are, however, inconsistent with a meta-analysis by Wahlbeck and colleagues [35], who reported lack of significant differences in dropout rates between typicals and atypicals other than clozapine. That meta-analysis included 163 randomized clinical trials, most of which were short-term, placebo-controlled trials. Importantly, the authors did not perform direct head-to-head comparisons of typicals and atypicals. When direct comparisons between typicals and atypicals were performed in another meta-analysis [36], the findings supported the superiority of atypicals over typicals on "treatment failure" that captured dropout rates and symptomatic relapse.

Consistent with prior research in usual care setting [37], we also found clozapine to be associated with the longest time to medication discontinuation, a finding that is congruent with clozapine's greater efficacy in meta-analytical reviews [8,9,11,35]. It is unclear, however, whether certain confounds are responsible for this finding. Unlike treatment with other antipsychotics, treatment with clozapine requires periodic blood monitoring to assess the risk of developing agranulocytosis. In addition to the likely initial selection of more adherent patients for treatment with clozapine in usual care, the frequent monitoring may help increase treatment duration with clozapine. Support for this hypothesis comes from randomized double-blind trials of clozapine versus olanzapine [38,39], in which the treatment groups did not significantly differ on dropout rates, possibly because the double-blind design required both treatment groups to undergo regular monitoring with blood tests.

There is growing interest in naturalistic studies that compare, among other variables, the differences in treatment duration between specific atypical and typical antipsychotics. Two very large, naturalistic, prospective, 3-year studies have been recently completed, reporting findings consistent with US-SCAP. One is the European Schizophrenia Outpatient Health Outcomes (EU-SOHO, N = 10,972), which was conducted in 10 European countries [30], and the other is the Inter-Continental SOHO (IC-SOHO, N = 7658), conducted in 27 other countries [31,32]. In addition, a recent naturalistic study of 2230 first-episode schizophrenia patients hospitalized in Finland [40] compared monotherapy treatment with various typical and atypical antipsychotics. The authors found the first- and second-generation antipsychotics to be highly heterogeneous groups with regard to effectiveness in a real-world setting, and reported that patients treated with clozapine or olanzapine had substantially lower discontinuation rates of their initial treatment than patients treated with haloperidol. In another recent study [41], our group conducted a post hoc analysis of time to all-cause medication discontinuation using data from a 1-year, randomized, open-label study of schizophrenia patients treated with olanzapine, risperidone, or typical antipsychotics. Findings from that post hoc analysis were almost identical to the present study. Namely, the 1-year survival rate for patients randomized to olanzapine was significantly higher than it was for patients randomized to typical antipsychotics of high-, medium-, or low-potency levels, and compared to perphenazine. The survival rate for patients randomized to risperidone was significantly higher than typicals of high- or medium-potency levels but did not significantly differ from olanzapine, from typicals of low potency, or from perphenazine.

Current findings need to be interpreted in light of their limitations. First, the non-randomized, naturalistic design of this study introduced potential selection bias, which cannot be completely eliminated despite our use of statistical adjustments for a set of socio-demographic and treatment characteristics. The treatment groups may have differed on unobserved (thus unadjusted), pre-existing measures, such as symptom severity at the time of medication initiation. As noted earlier, US-SCAP did not assess patients' symptomatology at the time of medication initiation, only at predetermined intervals. Other confounds may have also affected the findings. These include the potential period bias, episode bias, and the potential for preference of the sponsor's medication (olanzapine) over other atypicals by participating physicians. One cannot completely adjust for these biases using statistical models, especially not the potential "sponsor bias." It should be noted, however, that the treating clinicians in the US-SCAP study were not connected to the study and were generally unaware that particular patients were participating in the study. Furthermore, if treating clinicians were biased in favor of the sponsor and its drug, one would have expected clinicians to quickly switch patients to olanzapine from antipsychotics they were receiving at enrollment. This phenomenon was not found when assessed with a sensitivity analysis. It is also important to note that the 3 statistical approaches used in this study and the 5 additional sensitivity analyses did provide consistent findings. Furthermore, our core findings on differential effectiveness between typicals and atypicals are highly consistent with those found in prior RCTs [9-11,15,42], in retrospective claims database studies [4,21,43,44], and in naturalistic prospective studies [4,30-33,40].

A second study limitation is the small sample size of the ziprasidone treatment group, which limited the statistical power of the analyses. It should be noted, however, that the current findings are consistent with CATIE [14], in which the ziprasidone-treated patients had the shortest median time to all-cause discontinuation (3.5 months in the 18-month trial) among the 5 treatment comparators, and the highest rate of discontinuation rate for any cause (79%). These findings were not statistically significant, likely due to much-reduced statistical power (change from 85% power for comparisons versus other atypicals to 58% power versus ziprasidone).

Third, our study was not designed to assess reasons for initiation or discontinuation of the antipsychotic medications. Findings from recent research suggest, however, that in addition to patient preferences, antipsychotic medication efficacy [14,28], rather than safety or poor tolerability, is the primary reason for medication discontinuation in the treatment of schizophrenia.

And lastly, this study did not require patients (or treatment episodes) to be confined to antipsychotic monotherapy. Although our approach did not distinguish between the augmenting and augmented antipsychotics, it is important to note that augmentation roles change over time due to prevalent switching of antipsychotics, a phenomenon we captured in analyses with multiple treatment episodes. Furthermore, antipsychotic switching processes are often characterized by medication overlap with cross-titration, which amounts to short-term (and at times prolonged) antipsychotic polypharmacy, thus making true monotherapy treatment episodes relatively rare and possibly not representative of the complex treatment regimens in real-world practice. Most importantly, the current findings are highly consistent with RCTs [14,42] and 3 large naturalistic studies conducted outside of the U.S. that included only monotherapy-treated patients [30-32,40].

The strengths of this study lie primarily in its prospective, naturalistic, real-world, long-term perspective; its large sample size; the use of an observational approach without any planned treatment intervention; the ability to provide comparative data on a number of commonly used antipsychotics; the ability to generalize the findings to patients treated in large public systems of care across the United States; and the availability of medication information during hospitalizations, a type of data that is typically absent in claims databases. Further, this study offers comprehensive medication information because patients were also queried about use of psychiatric medications outside of their regular treatment site. When this occurred, systematic efforts were made to abstract this out-of-site information. Additional strengths of this study lie in its use of multiple statistical approaches and sensitivity analyses, to help check the robustness of the findings, and the ability to proactively address several analytical challenges that are inherent in analyses of naturalistic, longitudinal data.

## Conclusion

This study found that in the usual care of patients with schizophrenia spectrum disorders, there are significant differences in time to medication discontinuation for any cause between typical and atypical antipsychotics, regardless of the typical antipsychotic potency level, or when the typical antipsychotic is augmented with prophylactic anticholinergic treatment to ameliorate EPS. Findings suggest that naturalistic studies may complement RCTs and facilitate interpreting their meaning for usual practice in real-world settings. In addition, findings have shown that atypical antipsychotics are not a homogeneous group, because not all the studied atypicals differentiated significantly and consistently from typicals of various type or potency levels. And lastly, this study demonstrated the utility of a simple and global index- time to all-cause medication discontinuation – to help clinicians, patients, and policymakers measure discernable and important differences between treatment options in the usual care of patients with schizophrenia.

## Competing interests

Drs. Ascher-Svanum, Zhu, Faries, and Landbloom are employees of Eli Lilly and Company, Indianapolis, Indiana, and minor stockholders in that company.

Dr. Swartz has received research funding from Eli Lilly and Company, and consulting and educational fees from AstraZeneca Pharmaceuticals LP, Bristol-Myers Squibb, Eli Lilly and Company, and Pfizer Inc.

Dr. Swanson has received funding support from Eli Lilly and Company as a SCAP site investigator.

## Authors' contributions

• HAS conceived of the study, participated in its design, the analytical plan, the interpretation of the results, and drafted the manuscript.

• BZ and DF participated in the design of the study, the analytical plan, the interpretation of the results, performed the statistical analyses, and assisted drafting the manuscript.

• RL participated in the design of the study, the interpretation of the results, and assisted drafting of the manuscript.

• MS and JS were involved in data collection of US-SCAP as co-PIs at a study site, participated in the design of the study, the analytical plan, the interpretation of the results, and assisted drafting the manuscript.

## Pre-publication history

The pre-publication history for this paper can be accessed here:


